# Commentator Discussion: Fifteen years’ experience of direct bridge with venoarterial extracorporeal membrane oxygenation to heart transplantation

**DOI:** 10.1016/j.xjon.2024.09.018

**Published:** 2024-09-24

See Article page 286.

Presenter: Dr Mojgan Laali

**Dr Berhane Worku***(New York, NY)*. Thank you. I'd like to thank the American Association for Thoracic Surgery and the moderators for allowing me to speak today. And I'd like to congratulate you and your team for an excellent presentation and really adding significantly to our knowledge about extracorporeal membrane oxygenation (ECMO) abridged to transplant—a very large number of patients with really long follow-up. Now you're keeping the patients who are on ECMO preoperatively on ECMO postoperatively as a matter of protocol. But even in the first era, and I think even in the non-ECMO group, a fairly large number of patients—I think 40% to 50% were placed on ECMO postoperatively. And so, what do you think the reasons for that are? Is it recipient or donor characteristics? It still seems like a fairly significant number even before that.

**Figure:**
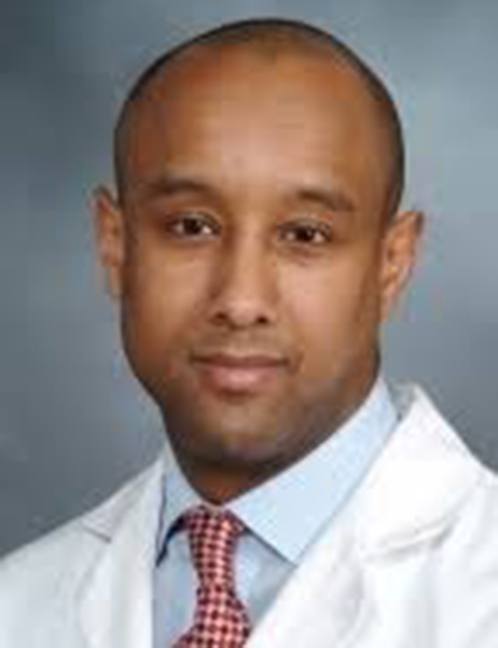

**DrMojgan Laali***(Paris, France)*. Yeah. It's both of them. In Europe donor age is varied. We go up to 65 years old, even 67 years old if the coronary angiography is normal and there are not any risk factors. So, donor age we know that every study is a risk factor of mortality. And we have primary graft dysfunction. It's 1 of the factors that cause primary graft dysfunction. So, for this reason—and on the other side we expand our recipients. We accept the patients that are refused in all part of the France in our center. By the way, we accept primary graft dysfunction. I presented a study that we have done in this cohort for primary graft dysfunction after transplantation, and we showed that there is not any significant difference for mortality. So, we see that with ECMO we can save the patients that the other center refused to transplant, yeah.

**Figure:**
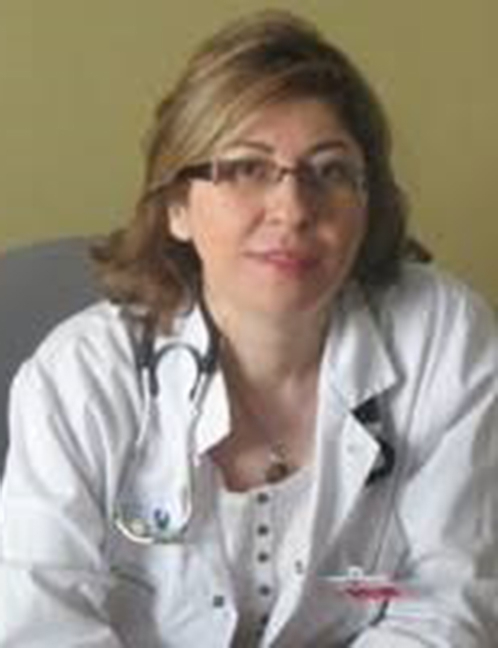

**Dr Worku**. Interesting. That's well said. And on that note, it seems that—well, you credit the improvement in outcomes over the later era in the ECMO group to liberal use postoperatively to peripheral cannulation. But as you've realized that outcomes after ECMO bridge to transplant are maintained, is it possible that also more liberal use preoperatively is contributing to this? Perhaps preoperatively putting patients on ECMO at an earlier stage in their using it more liberally preoperatively, has that contributed to improvements in outcomes? Do you have any information about these patients' presentations initially prior to cannulation?

**Dr Laali**. I think we are enough liberal to put ECMO before transplantation because one-third of our patients are on ECMO before transplantation and not most of them—the new cardiogenic shock. Some of them are patients known to the series. A quarter of the patients on ECMO are already known to the series. They are already listed for transplantation, and they arrive with decompensated cardiogenic shock. And if we saw that the drug—we have many drugs to use to stabilize their condition. We insert ECMO very early.

**Dr Worku**. Okay. And my last question. I'm not sure about the differences in allocation systems in different countries, but certainly, it seems that a lot of places have really escalated ECMO to its own tier or to really reduce wait list times. As the use of ECMO becomes more than a one-off and is, I think in your study almost 30% of patients go into transplant and on ECMO. And as the outcomes basically become equivalent to those who are not on ECMO, what do you think the implications for future allocation systems revisions are as this becomes commonplace?

**Dr Laali**. Yeah. We had exactly the same allocation system as, actually, you have in the United States, in fact, which was started in 2004. But we saw with these studies that there is overprioritization of patients on ECMO. So, the center that is not the program of ECMO, the patients on the list never have a chance to be transplanted. So, we changed the system to an allocation system by scoring. And with this system, we see that we can transplant the patient on ECMO because we have more longer time of prioritization on ECMO, which is 16 days, instead of 7 days that we had already. So, in this way, all the centers that don't have ECMO program, they can transplant their patient, and the center with a very high-volume ECMO program can transplant the patient too. But again, we have a shortage of programs, like everywhere. And for example, in this cohort, 400 patients were put in ECMO, and they are in the list for transplantation, but we have the possibility to transplant 317. So, there are 16 or 17 patients that weren't transplanted. Some of them, we put on lung assist device like left ventricular assist device or biventricular assist device and some of them died on ECMO. But we can save 370 patients, yeah.

**Dr Worku**. Well said. I'd like to thank the American Association for Thoracic Surgery and the moderators. Thank you.

**Dr Laali**. Thank you very much.

**Dr Worku**. Congratulations.

**Dr Laali**. Thank you.

**Unidentified Speaker 1**. All right. Thank you for showing your excellent reason. One of the emerging problems with the peripheral venoarterial ECMO is paralysis. The recent report from Maryland shows that up to 5% of people can develop paralysis, and that depends on the time too. I notice your time is only 4 days. The median time on the ECMO is only 4 days. But your program hasn't seen any paralysis with the peripheral venoarterial ECMO, and how do you prevent it, and how do you recognize it?

**Dr Laali**. The complication of the peripheral ECMO?

**Unidentified Speaker 1**. Yes.

**Dr Laali**. In the first era, we had more ECMO complications because we do surgical insertion. Now we do peripheral insertion and echocardiograph-guided, so there is no complication of infection or something like that. We always put retrograde perfusion. We use a 17 Fr cannula, not larger, and all of these factors save the complications. Yeah.

**Unidentified Speaker 1**. Yeah. The paralysis is the 1 that—

**Dr Laali**. We don't have so many.

**Unidentified Speaker 1**. You don't have that—

**Dr Laali**. No. No.

**Unidentified Speaker 1**. Well, thanks.

**Dr Worku**. Is that a unilateral, ipsilateral, or is—

**Unidentified Speaker 1**. No, ipsilateral.

**Dr Worku**. Is it like a paraplegia?

**Unidentified Speaker 1**. Ipsilateral. With data from ECMO—and I have 2 patient that have paralysis with ipsilateral, and then I saw the report from Maryland that show that up to 5% of these [crosstalk]—

**Dr Worku**. Is that femoral nerve injury or--?

**Unidentified Speaker 1**. What's that?

**Dr Worku**. You think it's femoral nerve injury or not?

**Unidentified Speaker 1**. No. It's bilateral paralysis, and it is due to spinal ischemia.

**Dr Laali**. No, I never saw that.

**Dr Worku**. Has anybody seen that? Anybody in the audience? Yes, you have.

**Unidentified Speaker 2**. Congratulations on your series, and the significant experience of having fresh heart transplants on ECMO. Can you tell us a little bit about how you managed the ECMO immediately after heart transplants as you're leaving in the operating room on ECMO? What's sort of your target flow? Are you allowing some flow through the heart to eliminate the risk of having clots develop in the freshly transplanted heart? And how are you managing anticoagulation? Are you putting them on anticoagulation, or are you taking them off the next day, or can you just tell us a little bit about that management?

**Dr Laali**. Yeah, okay.

**Unidentified Speaker 2**. I think it would be valuable.

**Dr Laali**. It depends on how allograft function. As I said, routinely, we keep ECMO, even if allograft functions normally. So, if allograft functions normally and we keep ECMO because it's in our policy, we put that on 2.5 L/minute, not more. It's enough for the patients. If we see that the allograft doesn't function anymore, suddenly we have a very important primary graft dysfunction, we keep ECMO on patient's needs for that for 4.5 L/minute or more, and we insert intra-aortic balloon pump at the same time to prevent complication of clot in the left ventricle. And we continue heparin 6 hours after operation if there is not any important bleeding, and we keep in time 2.5 longer than normal. Yeah. It's our policy.

**Unidentified Speaker 3**. Thank you very much.

**Dr Laali**. Thank you.

[applause]

## Conflict of Interest Statement

The authors reported no conflicts of interest.

The *Journal* policy requires editors and reviewers to disclose conflicts of interest and to decline handling or reviewing manuscripts for which they may have a conflict of interest. The editors and reviewers of this article have no conflicts of interest.

